# Non-invasive Detection of Exosomal MicroRNAs via Tethered Cationic Lipoplex Nanoparticles (tCLN) Biochip for Lung Cancer Early Detection

**DOI:** 10.3389/fgene.2020.00258

**Published:** 2020-03-20

**Authors:** Chang Liu, Eric Kannisto, Guan Yu, Yunchen Yang, Mary E. Reid, Santosh K. Patnaik, Yun Wu

**Affiliations:** ^1^Department of Biomedical Engineering, University at Buffalo – The State University of New York, Buffalo, NY, United States; ^2^Department of Thoracic Surgery, Roswell Park Comprehensive Cancer Center, Buffalo, NY, United States; ^3^Department of Biostatistics, University at Buffalo – The State University of New York, Buffalo, NY, United States; ^4^Department of Medicine, Roswell Park Comprehensive Cancer Center, Buffalo, NY, United States

**Keywords:** exosomes, circulating microRNAs, cancer biomarker, liquid biopsy, lung cancer

## Abstract

Circulating microRNAs carried by exosomes have emerged as promising diagnostic biomarkers for cancer because of their abundant amount and remarkable stability in body fluids. Exosomal microRNAs in blood are typically quantified using the RNA isolation-qRT-PCR workflow, which cannot distinguish circulating microRNAs secreted by cancer cells from those released by non-tumor cells, making it potentially less sensitive in detecting cancer-specific microRNA biomarkers. We have developed a sensitive and simple tethered cationic lipoplex nanoparticles (tCLN) biochip to detect exosomal microRNAs in human sera. The tCLN biochip allows the discrimination of tumor-derived exosomes from their non-tumor counterparts, and thus achieves higher detection sensitivity and specificity than qRT-PCR. We have demonstrated the clinical utility of the tCLN biochip in lung cancer diagnosis using sera from normal controls, therapy-naive early stage and late stage non-small cell lung cancer (NSCLC) patients. Total five microRNAs (miR-21, miR-25, miR-155, miR-210, and miR-486) were selected as the biomarkers. Each microRNA biomarker measured by tCLN assay showed higher sensitivity and specificity in lung cancer detection than that measured by qRT-PCR. When all five microRNAs were combined, the tCLN assay distinguished normal controls from all NSCLC patients with sensitivity of 0.969, specificity of 0.933 and AUC of 0.970, and provided much better diagnostic accuracy than qRT-PCR (sensitivity = 0.469, specificity = 1.000, AUC = 0.791). Remarkably, the tCLN assay achieved absolute sensitivity and specificity in discriminating early stage NSCLC patients from normal controls, demonstrating its great potential as a liquid biopsy assay for lung cancer early detection.

## Introduction

Lung cancer is the leading cause of cancer death in the United States. The 5 year survival rate is ∼55% for early stage lung cancer but only 5% for late stage lung cancer, reflecting the urgent need for effective screening and early detection methods (Siegel et al., 2019). Although low dose computed tomography (CT) is recommended as the screening test for patients at high risk of lung cancer, this test suffers a high false positive rate (> 95%) and radiation risk (Patz et al., 2014; Chudgar et al., 2015; Krantz and Meyers, 2015). Detecting circulating biomarkers in blood via liquid biopsies is a promising and patient-friendly strategy for lung cancer early detection.

Exosomes are small, nanosized vesicles released by all types of cells. They stably exist in various body fluids, such as blood and urine, in large quantities. Exosomes carry many molecular cargos including microRNAs, mRNAs, DNA fragments and proteins from cells of origin (Kalluri, 2016; Xu et al., 2018). Exosomes, especially those released by tumor cells, are actively involved in cancer development, metastasis and drug resistance (Kalluri, 2016; Whiteside, 2016; Xu et al., 2018). Therefore, exosomes are promising biomarkers for cancer diagnosis. Circulating microRNAs carried by exosomes have been demonstrated as potential biomarkers for lung cancer. For example, a panel of circulating microRNAs including miR-21, miR-223, miR-155, and miR-126 were identified as potent biomarkers for lung cancer in a recent meta-analysis study involving 6919 lung cancer patients and 7064 controls (Yang et al., 2017).

Well-established techniques, such as qRT-PCR, microarray and next generation sequencing, are typically used to characterize exosomal microRNAs. However, the RNA isolation procedure used in these techniques mixes exosomal microRNAs released by tumor cells with those derived from all other non-tumor cells, and therefore these techniques only measure total amount of exosomal microRNAs in blood, and cannot detect exosomal microRNAs specifically coming from tumors. Besides, the clinical utilities of these techniques are also limited by their tedious and time consuming procedures and high cost.

**FIGURE 1:**
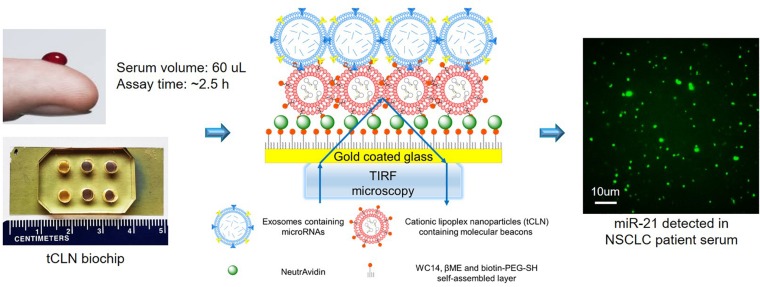
Overview of tCLN biochip and its detection mechanism. In the tCLN biochip, cationic lipoplex nanoparticles containing molecular beacons (CLN-MB) are tethered on the surface of a gold-coated cover glass. Exosomes are fused with CLN-MB through electrostatic interaction to form CLN-exosome complexes. Inside the complexes, MB hybridize to exosomal microRNAs and generate fluorescence signals, which are captured by total internal reflection fluorescence (TIRF) microscopy. The fluorescence intensity of each CLN-exosome complex is individually analyzed to measure the expression of exosomal microRNAs. The tCLN assay uses 60 uL serum and total assay time is about 2.5 h.

We have developed a tethered cationic lipoplex nanoparticles (tCLN) biochip that is capable of distinguishing cancer cell-derived exosomal microRNAs from normal cell-derived exosomal microRNAs, making it potentially more sensitive in cancer diagnosis than existing technologies, such as qRT-PCR (Wu et al., 2013). As shown in Figure 1, in tCLN biochip, cationic lipoplex nanoparticles containing molecular sensing probes, i.e., molecular beacons, are tethered on the surface of a gold-coated cover glass. After exosomes are applied, the electrostatic interaction between positively charged lipoplex nanoparticles and negatively charged exosomes makes these two types of nanoparticles fuse with each other and form the CLN-exosome complexes. Inside the CLN-exosome complexes, molecular beacons hybridize to microRNA targets and generate fluorescent signals, which are detected by total internal reflection fluorescence (TIRF) microscopy and used to quantify the expression of exosomal microRNAs. In tCLN assay, microRNAs are confined within the CLN-exosome complexes. This unique feature allows us to individually analyze the fluorescent signals from each CLN-exosome complex. During image analysis, we remove weak signals and only keep strong signals by using appropriate cut-off levels, therefore we are able to enlarge the differences between tumor cell-derived exosomes and normal cell-derived exosomes, and better distinguish tumor cell-derived exosomes from normal cell-derived exosomes. More detailed information about the detection mechanism, tCLN biochip characterization and image analysis can be found in our previous publication (Wu et al., 2013). In our feasibility study, we observed higher sensitivity of tCLN biochip in lung cancer diagnosis than qRT-PCR with two exosomal RNA biomarkers and sera from seven lung cancer patients and two normal controls (Wu et al., 2013). In this study, we have further evaluated the diagnostic performance of the tCLN biochip using a relative large number of non-small cell lung cancer (NSCLC) patients and normal controls. We have also expanded the biomarker panel to total five microRNAs and investigated their sensitivities and specificities in lung cancer detection. Our results have demonstrated that the tCLN biochip is a very promising liquid biopsy assay for lung cancer, offering both sensitivity and specificity above 0.93 with the panel of five microRNA biomarkers.

## Method

### Materials

1,2-Di-*O*-octadecenyl-3-trimethylammonium propane (DOT MA) and 1,2-distearoyl-sn-glycero-3-phosphoethanolamine-*N*-[biotinyl (polyethylene glycol)-2000] (ammonium salt) (Biotin-DSPE-PEG) were purchased from Avanti Polar Lipids, Inc. (Alabaster, AL, United States; 890898P and 880129P). Cholesterol and β-mercaptoethol (βME) were purchased from Sigma-Aldrich, Inc. (St. Louis, MO, United States; C3405 and M3148). 1-thiahexa (ethyleneoxide) lipidic anchor molecule WC14 [20-tetradacyloxy-3, 6, 9, 12, 15, 18, 22-heptapxahexa-tricontane-1-thiol] was a gift from Dr. James Lee’s lab at The Ohio State University. Biotinylated polyethylene glycol-thiol (biotin-PEG-SH) was purchased from Nanocs (Boston, MA; PG2-BNTH-600). (3-Mercaptopropyl) trimethoxysilane (MPS) was purchased from Sigma-Aldrich, Inc. (175617). SYLGARD^®^ 184 silicone elastomer kit (Polydimethylsiloxane, PDMS) was purchased from Dow Corning Corporation (Midland, MI, United States; 1696157). NeutrAvidin was purchased from Thermo Fisher Scientific, Inc. (Waltham, MA, United States; 31000). FAM or Cy5 labeled molecular beacons (MB) were synthesized by Sigma-Aldrich, Inc. See Supplementary Table S1 for details of molecular beacons.

### Preparation of Cationic Lipoplex Nanoparticles Containing Molecular Beacons (CLN-MB)

A mixture of FAM labeled MB and Cy5 labeled MB (mass ratio = 1:1) in 1X DPBS (Thermo Fisher Scientific; 14200075) was first mixed with the lipid mixture (DOTMA: Cholesterol: Biotin-DSPE-PEG = 49:49:2 molar ratio in ethanol) at the lipids to MB mass ratio of 12.5:1 and DPBS to ethanol volume ratio of 3:2. Then CLN-MB were prepared by injecting one part of the lipids/MB mixture into 9 parts of 1X DPBS followed by sonication at room temperature for 10 min.

### Fabrication of tCLN Biochip

Cover glasses (Fisher Scientific, Pittsburgh, PA, United States; 12-544D) were cleaned by ethanol and deionized (DI) water with 10 min sonication for each step. MPS was deposited onto clean cover glasses as the adhesion layer for Au coating in a vacuum chamber filled with evaporated MPS for 10 min. Excess MPS was removed by heating the cover glasses at 100°C for 30 min. A 15 nm Au thin film was deposited on the cover glasses by electron-beam evaporation (Kurt J. Lesker Company, Jefferson Hills, PA, United States) at a deposition rate of 5 Å/min.

To tether CLN-MB on the surface of the biochip, the Au coated cover glasses were first incubated with a mixture of WC14, βME and biotin-PEG-SH at molar ratio of 30:70:1 in ethanol overnight to form a self-assembled monolayer on Au surface. Then the biochip was incubated with 0.1 mg/mL NeutrAvidin for 30 min with gentle shaking. Excess NeutrAvidin was removed by three times of wash with DEPC treated water. Finally, the biochip was incubated with CLN-MB at MB concentration of 32 ug/mL for 30 min with gentle shaking to tether CLN-MB on the surface through biotin-avidin interaction. After excess CLN-MB were removed by three times of wash with DEPC treated water, the tCLN biochip was ready for use.

### Human Serum Samples

De-identified human serum samples and clinical data of 15 normal controls, 32 early stage (stage I/II) NSCLC patients and 32 late stage (stage III/IV) NSCLC patients were obtained from the Data Bank and BioRepository Shared Resource (DBBR) at Roswell Park Comprehensive Cancer Center (Buffalo, NY, United States). All patients were treatment naïve. Their baseline characteristics are provided in Supplementary Table S2. This study was approved by Institutional Review Boards at both University at Buffalo and Roswell Park Comprehensive Cancer Center.

### Exosome Isolation From Human Serum Samples

Total exosome isolation (from serum) reagent (Thermo Fisher Scientific; 4478360) was used to isolate exosome from serum samples following the manufacturer’s protocol with minor revision. Briefly, serum samples were centrifuged at 10,000 *g* for 30 min to remove debris. Then total exosome isolation reagent was added into serum samples at the reagent to serum volume ratio of 1:5. The mixture was incubated at 4°C for 30 min and centrifuged at 10,000 *g* for 10 min. The supernatant was removed and the exosome pellet was re-suspended in 1X DPBS at the serum to DPBS volume ratio of 2:1.

### Characterization of Exosomes

Exosomes were diluted 10,000 folds with 1X DPBS. Then the size, size distribution and number concentration of exosomes were measured by nanoparticle tracking analysis (NanoSight LM10, Malvern, Westborough, MA, United States). All measurements were performed at room temperature with the detection threshold of 6, the screen gain of 8, and the camera level at 14. The morphology of exosomes were characterized by Cryo Transmission Electron Microscopy (CryoTEM). Briefly, 5 μL exosome suspension in 1X DPBS was applied to glow discharged lacey carbon coated copper grids (400 mesh, Pacific Grid-Tech, San Francisco, CA, United States) and flash-frozen in liquid ethane using an automated vitrification device (FEI Vitrobot Mark IV, FEI, Hillsboro, OR, United States). The vitrified samples were transferred to a Gatan Cryo holder (Model 626.DH) and visualized in an FEI Tecnai G2 F20 ST TEM (FEI, Hillsboro, OR, United States). The quality of the exosomes was examined using Exo-Check Exosome Antibody Arrays (SBI system biosciences; EXORAY200A) following manufacturer’s protocol. The Exo-Check Exosome Antibody Arrays had total nine antibodies against eight known exosome markers (CD63, CD81, ALIX, FLOT1, ICAM1, EpCam, ANXA5, and TSG101) and one *cis-*Golgi marker (GM130) to monitor any cellular contamination.

### Characterization of Exosomal MicroRNAs by tCLN Biochip

To measure the levels of exosomal miR-21, miR-25, miR-155, miR-210, and miR-486 in human serum samples, 30 μL exosomes were added onto the tCLN biochip and incubated at 37°C for 2 h. TIRF microscopy (Eclipse Ti-E, Nikon Instruments, Inc.) was used to measure the fluorescence signals from MB in the FAM and Cy5 channels, respectively. Images were collected by an Andor iXon EMCCD camera with a 100X lens and exposure time of 200 ms. For each microRNA target, total 100 images were used to quantify its expression level following our previously reported protocol (Wu et al., 2013). Briefly, ImageJ was used to set the cut-off levels to remove signals from background and non-tumor cell derived exosomes. The sum intensity of remaining signals was calculated and used as the expression of microRNA target.

### Characterization of Serum MicroRNAs by qRT-PCR

The expression levels of miR-21, miR-25, miR-155, miR-210, and miR-486 in serum samples were measured using the RNA isolation-qRT-PCR workflow. Briefly, total RNA was extracted using miRCURY^TM^ RNA Isolation Kit – Biofluids (Exiqon; Woburn, MA, United States; 300112) from 100 uL serum. Then total RNA was reverse transcribed into cDNA using the Universal cDNA Synthesis Kit II (Exiqon; 203301). The qPCR amplification of cDNA was then performed using ExiLENT SYBR^®^ Green master mix (Exiqon; 203421). The primers for the five microRNAs were hsa-miR-21-5p LNA^TM^ PCR primer set (Exiqon; 204230), hsa-miR-25-5p LNA^TM^ PCR primer set (Exiqon; 204031), hsa-miR-155-5p LNA^TM^ PCR primer set (Exiqon; 204308), hsa-miR-210-3p LNA^TM^ PCR primer set (Exiqon; 204333) and hsa-miR-486-5p LNA^TM^ PCR primer set (Exiqon; 204001). The expression of microRNAs was normalized to miR-191 (hsa-miR-191-5p LNA^TM^ PCR primer set; Exiqon; 204306), which was used as the endogenous control in the corresponding samples (Hu et al., 2012; Zheng et al., 2013; Li et al., 2015). Relative gene expression values were reported by miR-191-normalized C_*q*_ values.

### Statistical Analysis

Binary logistic regression and receiver operating characteristic (ROC) analyses were performed to evaluate the sensitivity and specificity of each microRNA biomarker and all microRNA biomarkers combined in distinguishing normal controls from NSCLC patients. The pROC package in the R software was used (Robin et al., 2011). Cut-offs with the highest sum of sensitivity and specificity were first identified, and areas under the curve (AUC), sensitivity and specificity were calculated with these cut-offs. For two-group comparison, standard two-tailed *t*-tests were used, and a *p*-value of < 0.05 was used to deem significance.

## Results

Serum samples from 15 normal controls, 32 early stage (stage I/II) and 32 late stage (stage III/IV) NSCLC patients were used in this study. These patients were age, gender, and race matched. All NSCLC patients were treatment naïve (see Supplementary Table S2 for details about their baseline characteristics). Both adenocarcinoma and squamous cell carcinoma were included because they are two most common types of NSCLC, together constituting about 70% of NSCLC. Among total 64 NSCLC cases, 45 were adenocarcinoma and 19 were squamous cell carcinoma. Exosomes were isolated from 60 uL serum sample. The size, size distribution and number concentration were measured by nanoparticle tracking analysis. Supplementary Figure S1a showed typical size distributions of exosomes isolated from a normal control, a stage I NSCLC patient and a stage IV NSCLC patient. The average diameter of exosomes was 102 nm, and the average number concentration of exosomes was 5.25 × 10^12^ exosomes/mL. No significant difference was observed in the size and number concentration of exosomes between normal controls and NSCLC patients (Supplementary Figure S1b). The morphology of exosomes was observed using CryoTEM. Supplementary Figure S1c showed representative images of exosomes isolated from a stage IV NSCLC patient. Most exosomes showed single vesicle structures. The quality of exosomes was examined by the Exo-Check Exosome Antibody Arrays. Representative results of exosomes isolated from the serum of a stage IV NSCLC patient was shown in Supplementary Figure S1d. All exosome markers including CD63, CD81, ALIX, FLOT1, ICAM1, EpCam, ANXA5, and TSG101 were strongly expressed. Little expression of GM130 (a *cis*-Golgi marker) was observed. These results demonstrated that exosomes had high purity and no cellular contamination.

**FIGURE 2:**
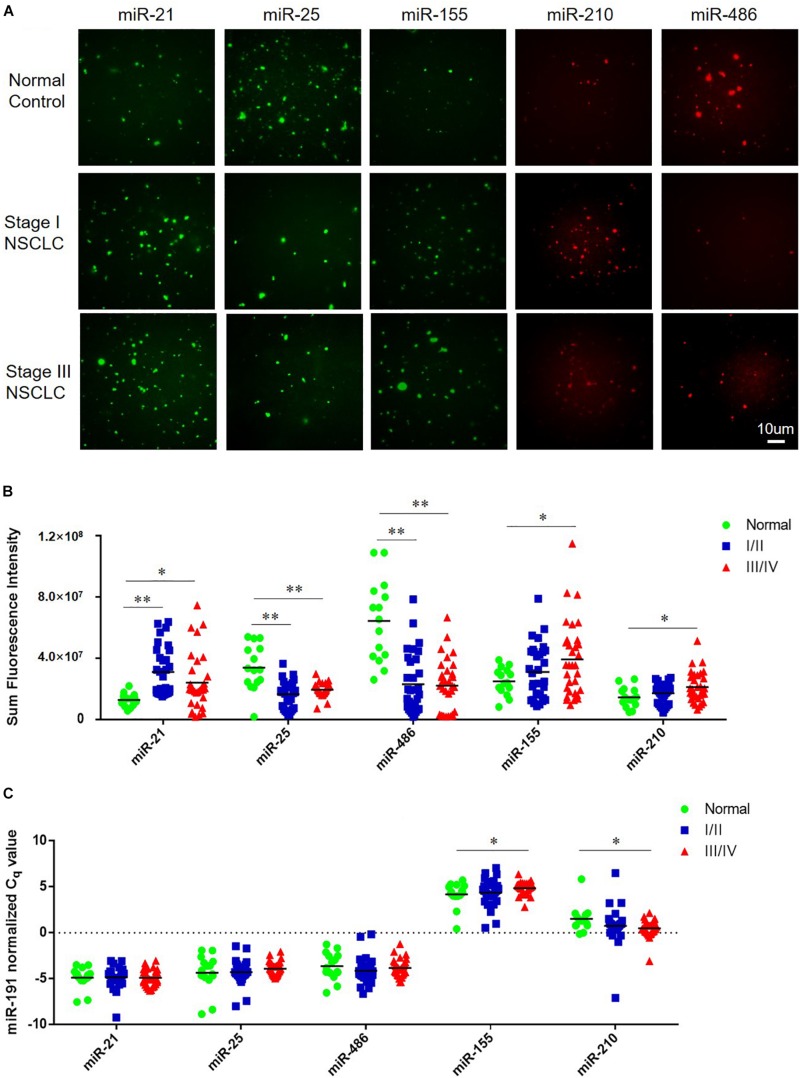
Expression of miR-21, miR-25, miR-155, miR-210, and miR-486 in serum samples from normal controls and NSCLC patients**. (A)** Representative images of miR-21, miR-25, miR-155, miR-210, and miR-486 obtained from tCLN assay using serum samples of a normal control, a Stage I NSCLC patient and a Stage III NSCLC patient. The expression of miR-21, miR-25, miR-155, miR-210, and miR-486 in serum samples measured by tCLN assay **(B)** and qRT-PCR **(C).** Serum samples from total 15 normal controls, 32 Stage I/II NSCLC patients and 32 stage III/IV NSCLC patients were included. Serum sample volume was 60 uL for tCLN assay and 100 uL for qRT-PCR. (^∗^*p* < 0.05; ^∗∗^*p* < 0.001).

The tCLN assay was used to measure the levels of miR-21, miR-25, miR-155, miR-210, and miR-486 based on the restored fluorescence intensity of molecular beacons. Figure 2A shows representative images of miR-21, miR-25, miR-155, miR-210, and miR-486 obtained with TIRF microscopy from a normal control, an early stage patient and a late stage patient. We observed significant differences in the expression of miR-21, miR-25, and miR-486 between normal controls and early stage NSCLC patients and between normal controls and late stage NSCLC patients (Figure 2B). Significant higher levels of miR-155 and miR-210 were detected in the serum samples from late stage NSCLC patients compared with normal controls, however, no significant difference in the expression of these two microRNAs was identified between normal controls and early stage NSCLC patients.

In order to demonstrate the advantages of tCLN assay over conventional methods, qRT-PCR was used to measure the expression of miR-21, miR-25, miR-155, miR-210, and miR-486 in serum samples from the same groups of normal controls and NSCLC patients. As shown in Figure 2C, we observed significant difference in the expression of miR-155 and miR-210 between normal controls and late stage NSCLC patients, but not between normal controls and early stage NSCLC patients. No significant difference was observed in the expression of miR-21, miR-25, and miR-486 between normal controls and NSCLC patients including both early stage and late stage patients.

**FIGURE 3:**
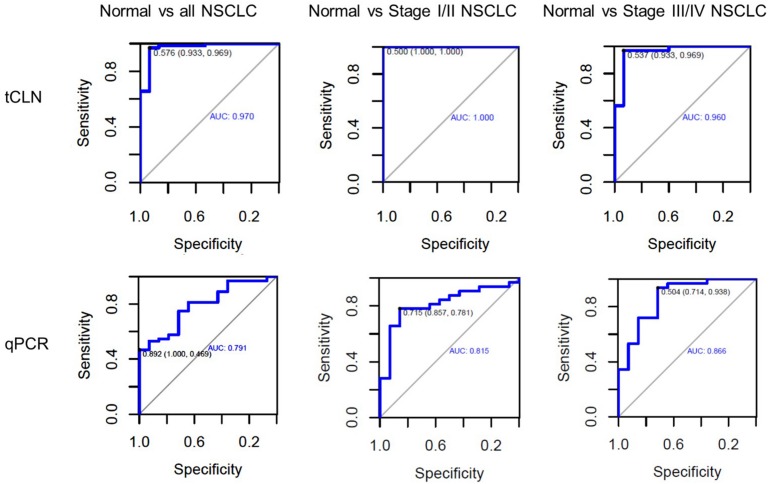
Diagnostic performance of a panel of microRNAs for lung cancer. ROC curves for a panel of 5 microRNAs consisting of miR-21, miR-25, miR-155, miR-210, and miR-486 measured by the tCLN biochip and qRT-PCR for lung cancer diagnosis. The detection sensitivity, specificity and AUC values were used to evaluate the diagnostic performances in distinguishing normal controls from all NSCLC cases, normal controls from early stage NSCLC patients, and normal controls from late stage NSCLC patients.

Binary logistic regression and ROC analyses were performed. The sensitivity, specificity and area under curve (AUC) values were calculated to investigate the diagnostic performance of each microRNA biomarker and the combined biomarker panel of all five microRNAs measured by tCLN assay and qRT-PCR (Supplementary Figure S2 and Figure 3). As summarized in Table 1, for each microRNA biomarker, the tCLN assay showed overall higher diagnostic accuracy, i.e., higher sensitivity, specificity and AUC values than qRT-PCR. For example, the miR-21 measured by tCLN assay distinguished normal controls from NSCLC patients with sensitivity of 0.828, specificity of 0.867 and AUC of 0.839, which were much higher than that measured by qRT-PCR (sensitivity of 0.453, specificity of 0.786 and AUC of 0.587). When all five microRNA biomarkers were combined, the tCLN assay showed sensitivity of 0.969 and specificity of 0.933 in distinguishing normal controls from all NSCLC patients (AUC = 0.970), while qRT-PCR only had sensitivity of 0.469 and specificity of 1.000 in detecting NSCLC (AUC = 0.791) (Figure 3). More importantly, the tCLN assay provided absolute sensitivity and specificity in differentiating early stage NSCLC from normal controls (AUC = 1.000), however, qRT-PCR only showed moderate diagnostic performance with sensitivity of 0.781, specificity of 0.578 and AUC of 0.815. These results demonstrated that the tCLN assay is a more sensitive and specific test than qRT-PCR in lung cancer diagnosis, especially for lung cancer early detection.

**TABLE 1:** Sensitivity, specificity and AUC of each biomarker and combined biomarkers in lung cancer diagnosis.

Assay	Biomarker	Normal vs. all NSCLC			Normal vs. early stage NSCLC			Normal vs. late stage NSCLC		
		SEN	SPE	AUC	SEN	SPE	AUC	SEN	SPE	AUC
**tCLN**	miR-21	0.828	0.867	0.839	0.938	0.867	0.943	0.688	0.933	0.734
	miR-25	0.875	0.800	0.863	0.844	0.800	0.856	0.906	0.800	0.870
	miR-155	0.406	1.000	0.627	0.375	1.000	0.585	0.438	1.000	0.668
	miR-210	0.719	0.667	0.670	0.688	0.667	0.622	0.750	0.667	0.719
	miR-486	0.734	0.933	0.910	0.719	0.933	0.898	0.844	0.867	0.923
	All biomarkers	0.969	0.933	0.970	1.000	1.000	1.000	0.969	0.933	0.960
**qRT-PCR**	miR-21	0.453	0.786	0.587	0.469	0.714	0.558	0.562	0.786	0.616
	miR-25	0.719	0.500	0.535	0.781	0.500	0.561	0.812	0.357	0.509
	miR-155	0.578	0.643	0.567	0.125	1.000	0.493	0.719	0.643	0.641
	miR-210	0.578	0.857	0.718	0.781	0.643	0.690	0.625	0.857	0.746
	miR-486	0.828	0.500	0.634	0.844	0.500	0.674	0.812	0.500	0.594
	All biomarkers	0.469	1.000	0.791	0.781	0.857	0.815	0.938	0.714	0.866

*SEN, sensitivity; SPE, specificity; AUC, area under the curve.*

## Discussion and Conclusion

Early detection is the only effective way to improve the overall survival of lung cancer. Only 16% of lung cancer cases are diagnosed at an early stage. Effective screening and early detection methods are urgently needed. Liquid biopsy that detects circulating cancer biomarkers may be a potent solution to this unmet need. Circulating microRNAs in blood have been demonstrated as promising biomarkers for lung cancer diagnosis. Results from recent meta-analyses of more than 100 studies showed that circulating microRNAs, such as miR-21, miR-155 and miR-126, have good diagnostic performance in lung cancer with both sensitivity and specificity above 0.8 (Yang et al., 2017; Yu et al., 2018, 2019). And the diagnostic accuracy can be further improved when a panel of microRNAs are used as the combined biomarkers.

Currently the RNA isolation-qRT-PCR workflow is the most widely used method to measure the levels of circulating microRNAs in blood. Next generation sequencing and microarray are alternative technologies that enable microRNA profiling and the identification of new microRNA targets. Recently, many new biosensing technologies, such as localized surface resonance plasmon (LSRP) biosensor (Joshi et al., 2015), microfluidic device (Taller et al., 2015), molecular beacons based assays (Lee et al., 2015; Lee et al., 2016) and electrochemical biosensors (Goda et al., 2012; Boriachek et al., 2018; Zhang et al., 2018), have been developed to offer sensitive, simple and fast detection of exosomal microRNAs. However, these technologies are not able to separate exosomal microRNAs derived from tumor cells from those released by normal cells, and thus may have limited diagnostic sensitivity. We have developed an immuno-biochip that uses antibodies to capture exosomes expressing tumor-associated proteins to improve the separation of cancer cell-derived exosomes from normal cell-derived exosomes (Yang et al., 2018). However, the immuno-biochip only measures the expression of microRNAs in a subpopulation of exosomes, i.e., exosomes carrying tumor-associated proteins, and cannot measure the levels of microRNAs from all tumor cell-derived exosomes. The tCLN biochip evaluated in this study provides the unique capability of detecting all exosomal microRNAs released from tumor cells but not normal cells.

We used both the tCLN biochip and qRT-PCR to measure the levels of miR-21, miR-25, miR-155, miR-210 and miR-486 in serum samples from 15 normal controls, 32 early stage and 32 late stage NSCLC patients (Figure 2). These five microRNAs were selected because they were reported in at least 5 different studies as promising diagnostic biomarkers for lung cancer (Yang et al., 2017; Yu et al., 2018, 2019; Li et al., 2019). Many microRNAs have been reported to be potential circulating biomarkers for lung cancer. However, the use of different patient cohorts, profiling platforms, normalization strategies, and analysis methods has contributed to the lack of a strong consensus among these microRNA biomarker studies. Therefore, we reviewed recent literature with a focus on meta-analyses of multiple studies and selected five microRNA biomarkers based on the consensus among various studies and their diagnostic performances in lung cancer. With tCLN biochip, we observed significantly increased expression of miR-21 and decreased expression of miR-25 and miR-486 in serum samples from both early stage and late stage NSCLC patients compared with normal controls. However, qRT-PCR did not show any significant differences in the expression of miR-21, miR-25 and miR-486 between normal controls and NSCLC patients, suggesting that tCLN biochip is more sensitive than qRT-PCR in cancer diagnosis. Both tCLN biochip and qRT-PCR detected significant differences in the expression of miR-155 and miR-210 between late stage NSCLC patients and normal controls. The levels of miR-155 and miR-210 showed no significant differences between early stage NSCLC patients and normal controls, suggesting that these two microRNAs might be biomarkers for NSCLC staging. Binary logistic regression and ROC analyses were performed to compare the detection sensitivity and specificity of tCLN assay with qRT-PCR. As summarized in Table 1 and Supplementary Figure S2, for each microRNA, tCLN assay showed overall higher sensitivity and specificity in distinguishing NSCLC patients from normal controls. Among these five microRNAs, for tCLN biochip, miR-486 showed the highest AUCs in distinguishing normal controls from all NSCLC cases and from late stage NSCLC patients, and miR-21 showed the highest AUC in discriminating normal controls from early stage NSCLC patients. For qRT-PCR, miR-210 was the best biomarker that had highest AUCs in differentiating NSCLC cases, both early stage and late stage, from normal controls. As shown in Table 1 and Figure 3, the diagnostic accuracy of both assays was further improved when all biomarkers were combined. With a panel of five microRNA biomarkers, the tCLN biochip showed much better diagnostic performance than qRT-PCR, with sensitivity of 0.969, specificity of 0.933 and AUC of 0.970 in distinguishing NSCLC from normal controls. Remarkably, the tCLN biochip showed absolute sensitivity and specificity (AUC = 1.000) in differentiating early stage NSCLC patients from normal controls, demonstrating its great potential in lung cancer early detection.

Lastly, we note that although the expression of many circulating microRNAs was reported to be dysregulated in blood, the direction of dysregulation was not quite consistent across different studies. MiR-21 is so far the only microRNA consistently reported to be up-regulated in blood by more than 15 studies (Yang et al., 2017; Yu et al., 2018, 2019). For other four microRNAs, both up- and down-regulation in their expression were reported (Yang et al., 2017; Yu et al., 2018, 2019). In our study, results from tCLN assay agreed well with the findings from majority of studies. For example, we observed significantly up-regulated miR-21 and down-regulated miR-486 expression in serum samples from NSCLC patients, which were consistent with most of the studies. However, qRT-PCR detected little difference in the expression of miR-21, miR-25, and miR-486 between NSCLC patients and normal controls. The discrepancy between our qRT-PCR results and other reports may be caused by small patient number, different patient characteristics, sample type (serum vs. plasma), and chemical reagents used in RNA isolation and qRT-PCR.

In summary, we have developed the tCLN biochip that enables the specific detection of tumor-derived exosomal microRNAs and thus provides high sensitivity and specificity for cancer diagnosis. The diagnostic performance of tCLN biochip in lung cancer was evaluated using five microRNAs as biomarkers. The tCLN biochip showed absolute sensitivity and specificity in distinguishing early stage NSCLC patients from normal controls, and > 0.93 sensitivity and specificity in distinguishing all NSCLC cases from normal controls, which were much higher than qRT-PCR.

In the future, we will further improve the sensing performance of the tCLN biochip in order to handle more complex biological fluids, such as whole blood. Because CLN have positive charges, in addition to exosomes, they interact with all negatively charged components in the blood, such as proteins and microvesicles, which affects the detection sensitivity. To address this challenge, we can integrate a microfluidic-based exosome isolation device with the tCLN biochip. Pure exosomes will first be isolated from biological fluids using the microfluidic device, and exosomal microRNAs are then detected by the tCLN assay. Currently, the tCLN assay uses TIRF microscopy-based imaging to allow the analysis of individual CLN-exosome complexes. Portable, small footprint and low-cost TIRF microscopy systems, which are available on the market, may be integrated with tCLN biochip to enable point-of-care testing and to facilitate the clinical translation. Lastly, the tCLN assay needs to be further validated to justify its clinical utility. In this study, we used well-defined patient cohorts with known disease status to train the tCLN assay, to establish appropriate operating parameters (such as sample volume, incubation time and cut-off levels used in image analysis), and to demonstrate the potential of tCLN assay in cancer early detection. However, we recognize that the sample size is small and the clinical outcome is not certain. The performance of the tCLN assay and the diagnostic values of the five exosomal microRNAs need to be further validated in blind studies using large cohorts of normal controls, patients at high risk of lung cancer, and NSCLC patients. We will also explore the applications of tCLN biochip in screening, early detection, treatment response monitoring and prognosis of other types of cancer.

## Data Availability Statement

All datasets generated for this study are included in the article/Supplementary Material.

## Ethics Statement

The studies involving human participants were reviewed and approved by the Institutional Review Boards at both University at Buffalo and Roswell Park Comprehensive Cancer Center. The patients/participants provided their written informed consent to participate in this study.

## Author Contributions

YW conceived and designed the study. CL and EK conducted the experiments and collected experimental data. CL, EK, GY, YY, SP, and YW analyzed and interpreted the data. MR and SP assisted with serum samples procurement for this study. YW and CL prepared the manuscript with contributions from all other authors.

## Conflict of Interest

The authors declare that the research was conducted in the absence of any commercial or financial relationships that could be construed as a potential conflict of interest.
