# Supplementary material for: Non-invasive Detection of Exosomal MicroRNAs via Tethered Cationic Lipoplex Nanoparticles (tCLN) Biochip for Lung Cancer Early Detection
Source: Front Genet. 2020 Mar 20;11:258. doi: 10.3389/fgene.2020.00258 (PMC7100709; doi:10.3389/fgene.2020.00258)
Supplement: Supplementary file 1 [file Table_1.docx]

Supplementary Material

Noninvasive detection of exosomal microRNAs via tethered cationic lipoplex nanoparticles (tCLN) biochip for lung cancer early detection

**Chang Liu^1^, Eric Kannisto^2^, Guan Yu^3^, Yunchen Yang^1^, Mary E. Reid^4^, Santosh K. Patnaik^2^, Yun Wu^1,*^**

^1^ Department of Biomedical Engineering, University at Buffalo, The State University of New York, 332 Bonner Hall, Buffalo, NY 14260, United States

^2^ Department of Thoracic Surgery, Roswell Park Comprehensive Cancer Center, Elm and Carlton Street, Buffalo, NY 14263, United States

^3^ Department of Biostatistics, University at Buffalo, The State University of New York, 710 Kimball Tower, Buffalo, NY 14214, United States

^4^ Department of Medicine, Roswell Park Comprehensive Cancer Center, Elm and Carlton Street, Buffalo, NY 14263, United States

*** Correspondence:**Yun Wu, Ph.D.

Email: [ywu32@buffalo.edu](mailto:ywu32@buffalo.edu)

Phone: 716-645-8498

Fax: 716-645-2207

Keywords: exosome, circulating microRNA, liquid biopsy, lung cancer

**Supplementary Table S1. Sequence of molecular beacons.** Total 5 molecular beacons specific to miR-21, miR-25, miR-155, miR-210 and miR-486 were used in this study. Locked nucleic acids were included to enhance the hybridization efficiency between molecular beacons and target microRNAs.

| **Molecular Beacon ID** | **Sequence** |
| --- | --- |
| MB-miR-21 | [6FAM]CGCGATC[+T]CA[+A]CA[+T]CA[+G]TC[+T]GA[+T]AA [+G]CTAGATCGCG[BHQ1] |
| MB-miR-25 | [6FAM]CGCGATC[+T]CA[+G]AC[+C]GA[+G]AC[+A]AG[+T]GC  [+A]ATGGATCGCG[BHQ1] |
| MB-miR-155 | [6FAM]CGCGATC[+A]CC[+C]CT[+A]TC[+A]CG[+A]TT[+A]GC  [+A]TTAAGATCGCG[BHQ1] |
| MB-miR-210 | [Cyanine5]CGCGATC[+T]CA[+G]CC[+G]CT[+G]TC[+A]CA[+C]  GC[+A]CAGGATCGCG[BHQ3] |
| MB-miR-486 | [Cyanine5]CGCGATC[+C]TC[+G]GG[+G]CA[+G]CT[+C]AG[+T]  AC[+A]GGAGATCGCG[BHQ3] |

[+A], [+T], [+G], [+C]: locked nucleic acids.

**Supplementary Table S2.** **Baseline** **characteristics of normal controls and NSCLC patients.**

| **ID** | **Morphology** | **Race** | **Gender** | **AgeCat** | **Stage** |
| --- | --- | --- | --- | --- | --- |
| 1 | Normal | White | F | 55-59 |  |
| 2 | Normal | White | F | 75-79 |  |
| 3 | Normal | White | F | 60-64 |  |
| 4 | Normal | White | M | 50-54 |  |
| 5 | Normal | White | F | 65-69 |  |
| 6 | Normal | White | F | 55-59 |  |
| 7 | Normal | White | F | 65-69 |  |
| 8 | Normal | White | M | 55-59 |  |
| 9 | Normal | White | F | 60-64 |  |
| 10 | Normal | White | M | 60-64 |  |
| 11 | Normal | White | M | 60-64 |  |
| 12 | Normal | White | M | 65-69 |  |
| 13 | Normal | White | M | 65-69 |  |
| 14 | Normal | White | M | 50-54 |  |
| 15 | Normal | White | F | 75-79 |  |
| 16 | Adenocarcinoma | Black | M | 50-54 | 1A |
| 17 | Adenocarcinoma | White | M | 70-74 | 1A |
| 18 | Adenocarcinoma | White | M | 70-74 | 1A |
| 19 | Adenocarcinoma | White | F | 70-74 | 1A |
| 20 | Adenocarcinoma | White | F | 70-74 | 1A |
| 21 | Adenocarcinoma | White | F | 50-54 | 1A |
| 22 | Adenocarcinoma | White | F | 70-74 | 1A |
| 23 | Squamous cell carcinoma | White | F | 65-69 | 1A |
| 24 | Adenocarcinoma | White | F | 65-69 | 1A |
| 25 | Squamous cell carcinoma | White | M | 60-64 | 1A |
| 26 | Adenocarcinoma | White | F | 70-74 | 1A |
| 27 | Adenocarcinoma | White | F | 55-59 | 1A |
| 28 | Adenocarcinoma | White | F | 60-64 | 1A |
| 29 | Adenocarcinoma | White | M | 65-69 | 1A |
| 30 | Adenocarcinoma | White | F | 55-59 | 1B |
| 31 | Adenocarcinoma | White | F | 60-64 | 1B |
| 32 | Adenocarcinoma | White | F | 55-59 | 1B |
| 33 | Squamous cell carcinoma | White | F | 50-54 | 1B |
| 34 | Adenocarcinoma | White | F | 55-59 | 1B |
| 35 | Adenocarcinoma | White | F | 60-64 | 1B |
| 36 | Squamous cell carcinoma | White | M | 60-64 | 2A |
| 37 | Adenocarcinoma | White | F | 55-59 | 2A |
| 38 | Adenocarcinoma | White | M | 65-69 | 2A |
| 39 | Squamous cell carcinoma | White | M | 55-59 | 2A |
| 40 | Adenocarcinoma | White | F | 65-69 | 2A |
| 41 | Adenocarcinoma | White | M | 45-49 | 2B |
| 42 | Squamous cell carcinoma | White | M | 65-69 | 2B |
| 43 | Squamous cell carcinoma | White | M | 75-79 | 2B |
| 44 | Squamous cell carcinoma | White | M | 75-79 | 2B |
| 45 | Adenocarcinoma | White | F | 55-59 | 2B |
| 46 | Adenocarcinoma | White | F | 70-74 | 2B |
| 47 | Adenocarcinoma | White | M | 60-64 | 2B |
| 48 | Adenocarcinoma | White | M | 70-74 | 3A |
| 49 | Squamous cell carcinoma | White | F | 70-74 | 3A |
| 50 | Squamous cell carcinoma | White | F | 70-74 | 3A |
| 51 | Adenocarcinoma | White | F | 60-64 | 3A |
| 52 | Squamous cell carcinoma | White | M | 75-79 | 3A |
| 53 | Squamous cell carcinoma | White | F | 60-64 | 3A |
| 54 | Squamous cell carcinoma | White | M | 70-74 | 3A |
| 55 | Adenocarcinoma | White | M | 65-69 | 3A |
| 56 | Adenocarcinoma | White | M | 55-59 | 3A |
| 57 | Adenocarcinoma | White | F | 65-69 | 3A |
| 58 | Squamous cell carcinoma | White | M | 60-64 | 3B |
| 59 | Adenocarcinoma | White | M | 65-69 | 3B |
| 60 | Adenocarcinoma | White | M | 60-64 | 3B |
| 61 | Squamous cell carcinoma | White | F | 65-69 | 3B |
| 62 | Adenocarcinoma | White | M | 55-59 | 3B |
| 63 | Squamous cell carcinoma | White | F | 55-59 | 3B |
| 64 | Adenocarcinoma | White | M | 55-59 | 4 |
| 65 | Adenocarcinoma | White | F | 60-64 | 4 |
| 66 | Squamous cell carcinoma | White | M | 55-59 | 4 |
| 67 | Adenocarcinoma | White | M | 50-54 | 4 |
| 68 | Adenocarcinoma | White | F | 40-44 | 4 |
| 69 | Adenocarcinoma | White | F | 75-79 | 4 |
| 70 | Adenocarcinoma | White | F | 70-74 | 4 |
| 71 | Adenocarcinoma | White | M | 50-54 | 4 |
| 72 | Squamous cell carcinoma | White | M | 70-74 | 4 |
| 73 | Adenocarcinoma | White | M | 55-59 | 4 |
| 74 | Squamous cell carcinoma | White | F | 55-59 | 4 |
| 75 | Adenocarcinoma | White | M | 75-79 | 4 |
| 76 | Adenocarcinoma | White | F | 70-74 | 4 |
| 77 | Adenocarcinoma | White | F | 65-69 | 4 |
| 78 | Adenocarcinoma | White | M | 50-54 | 4 |
| 79 | Adenocarcinoma | White | F | 75-79 | 4 |

**Supplementary Figure S1.** **Characterization of exosomes.** (a) Representative size distributions of exosomes isolated from serum samples from a normal control, a stage I NSCLC patient, and a stage IV NSCLC patient. (b) No significant difference was observed in exosome concentration between normal controls, early stage and late stage NSCLC patients. (c) CryoTEM image of exosomes isolated from the serum of a stage IV NSCLC patient. (d) Quality of exosomes was examined by Exo-Check Exosome Antibody Arrays. Representative result of exosomes isolated from the serum of a stage IV NSCLC patient. All exosome markers (CD63, CD81, ALIX, FLOT1, ICAM1, EpCam, ANXA5 and TSG101) were observed. No significant expression of GM130 (a cis-Golgi marker) was observed. These results indicated that exosome samples had high purity and cellular contamination was minimal.

**
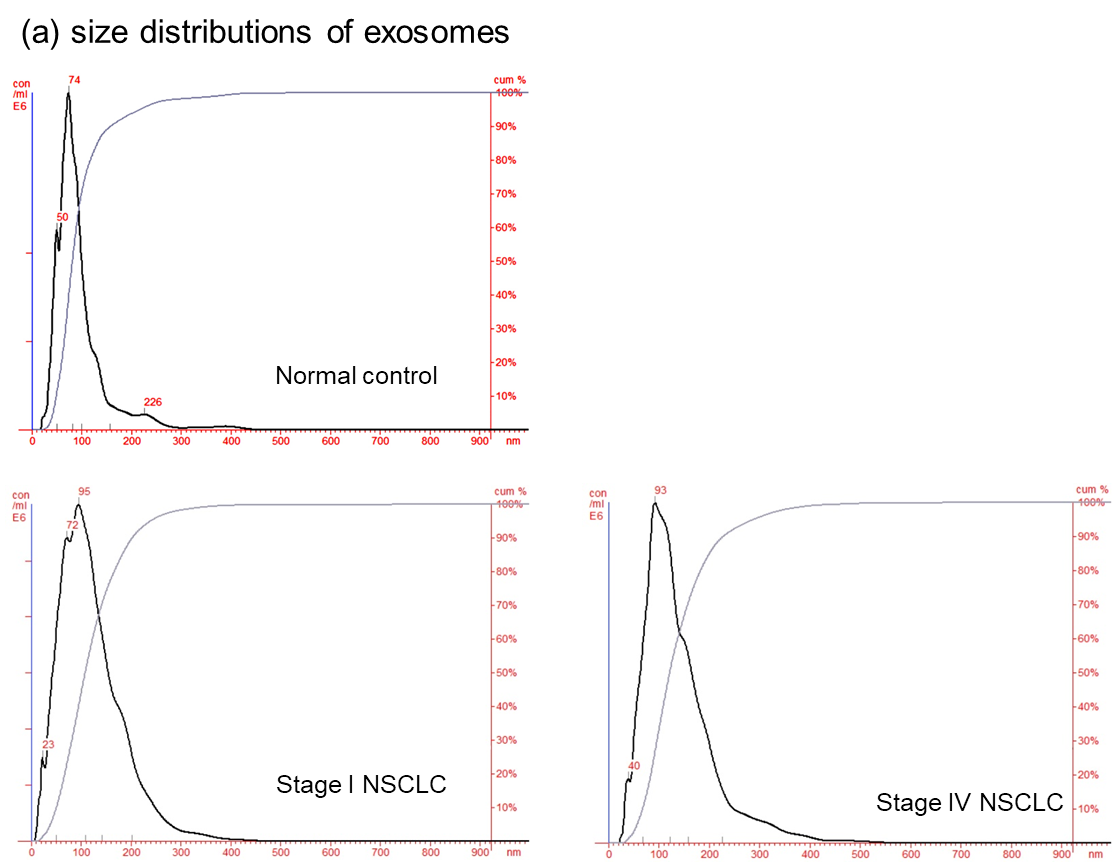
**

**
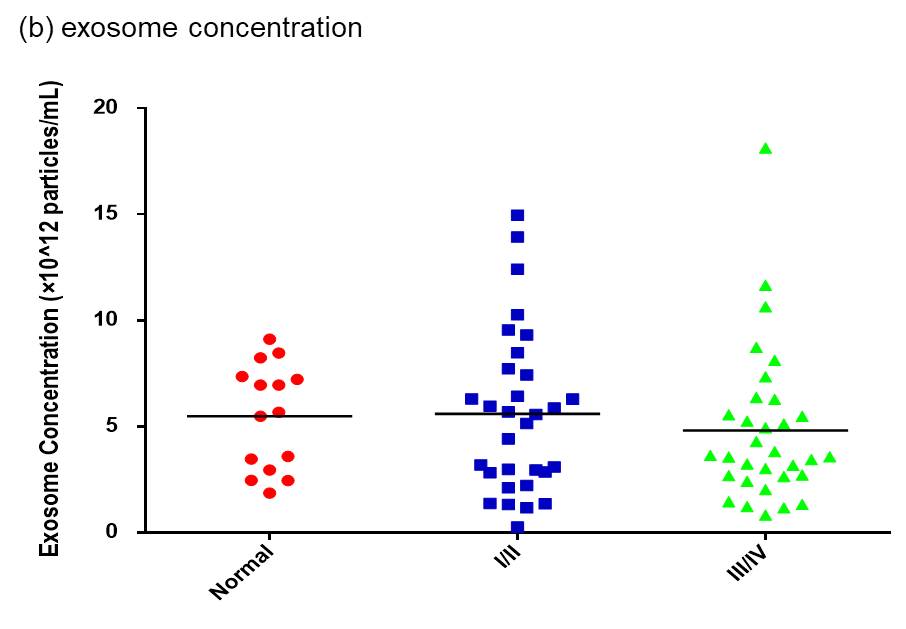
**

**
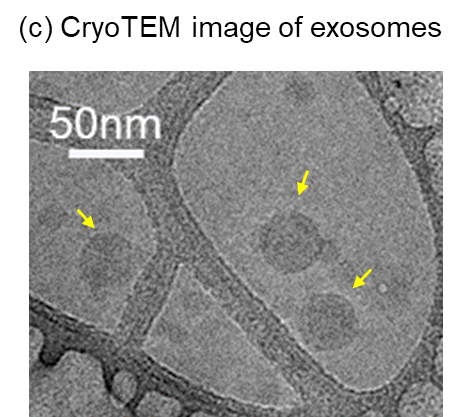
**

**
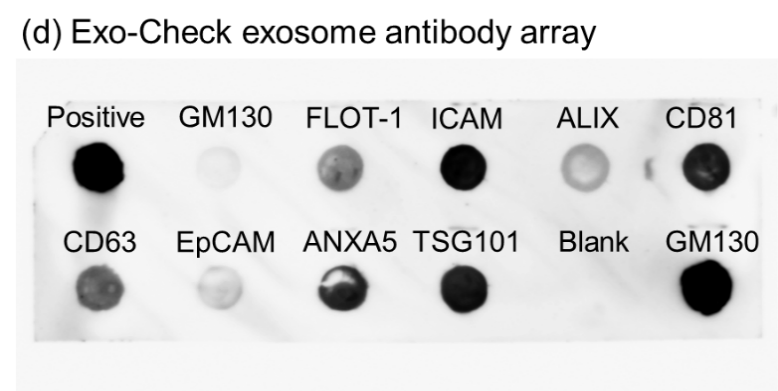
**

**Supplementary Figure S2. Diagnostic performance of 5 microRNAs for lung cancer.** ROC curves for (a) miR-21, (b) miR-25, (c) miR-155, (d) miR-210 and (e) miR-486 measured by the tCLN biochip and qRT-PCR for lung cancer diagnosis. The detection sensitivity, specificity and AUC values were used to evaluate the diagnostic performances in distinguishing normal controls from all NSCLC cases, normal controls from early stage NSCLC patients, and normal controls from late stage NSCLC patients.

**
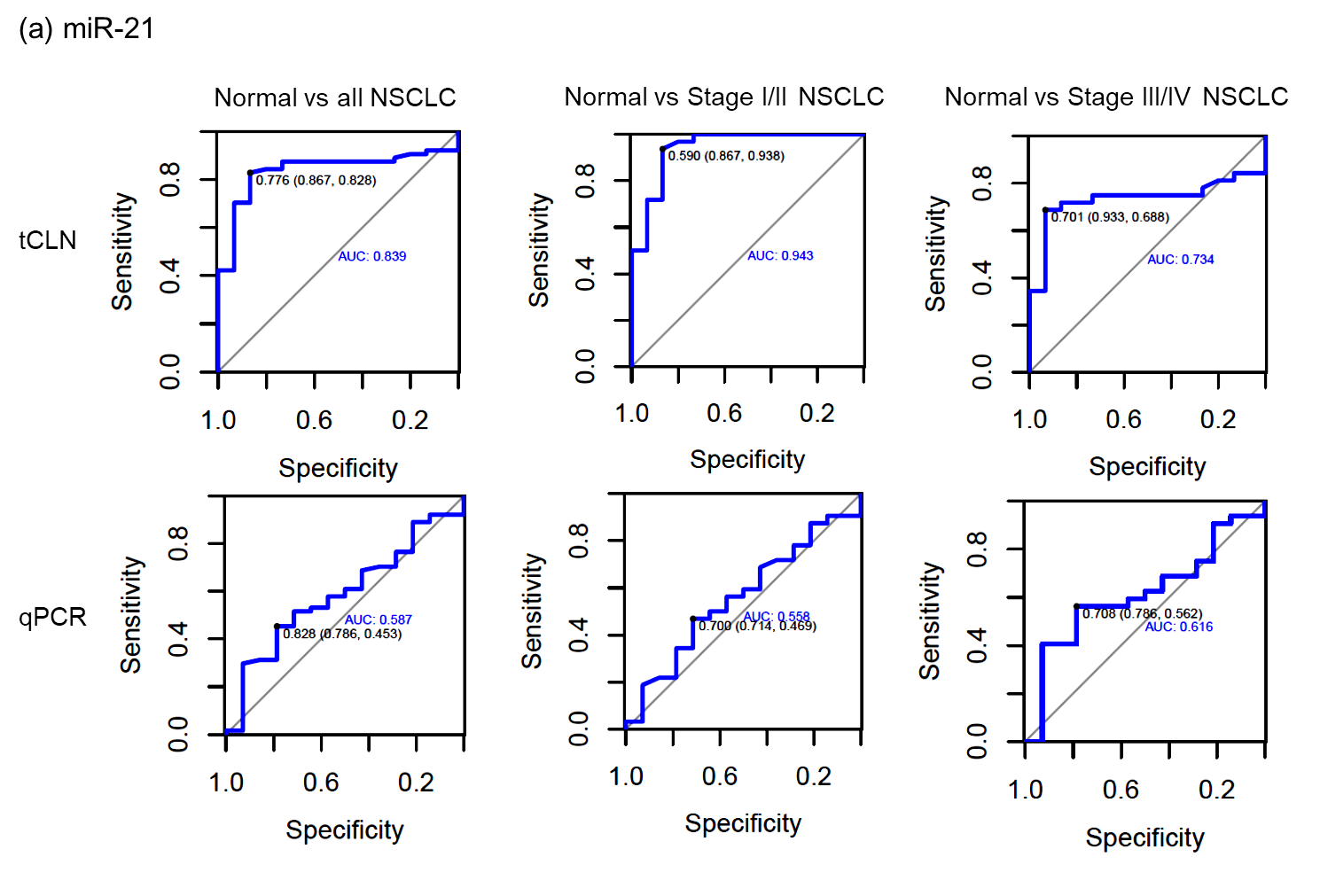
**

**
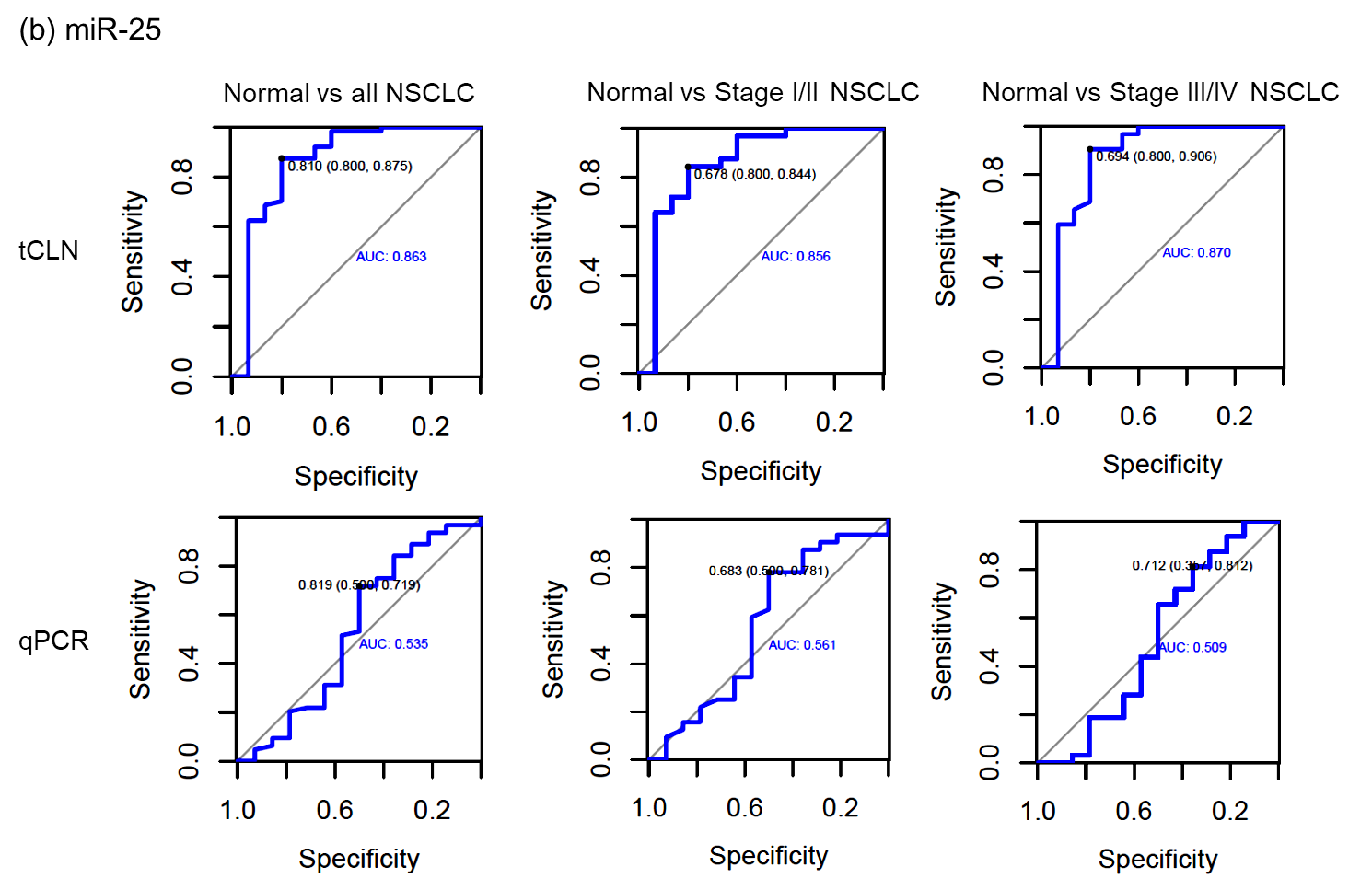
**

**
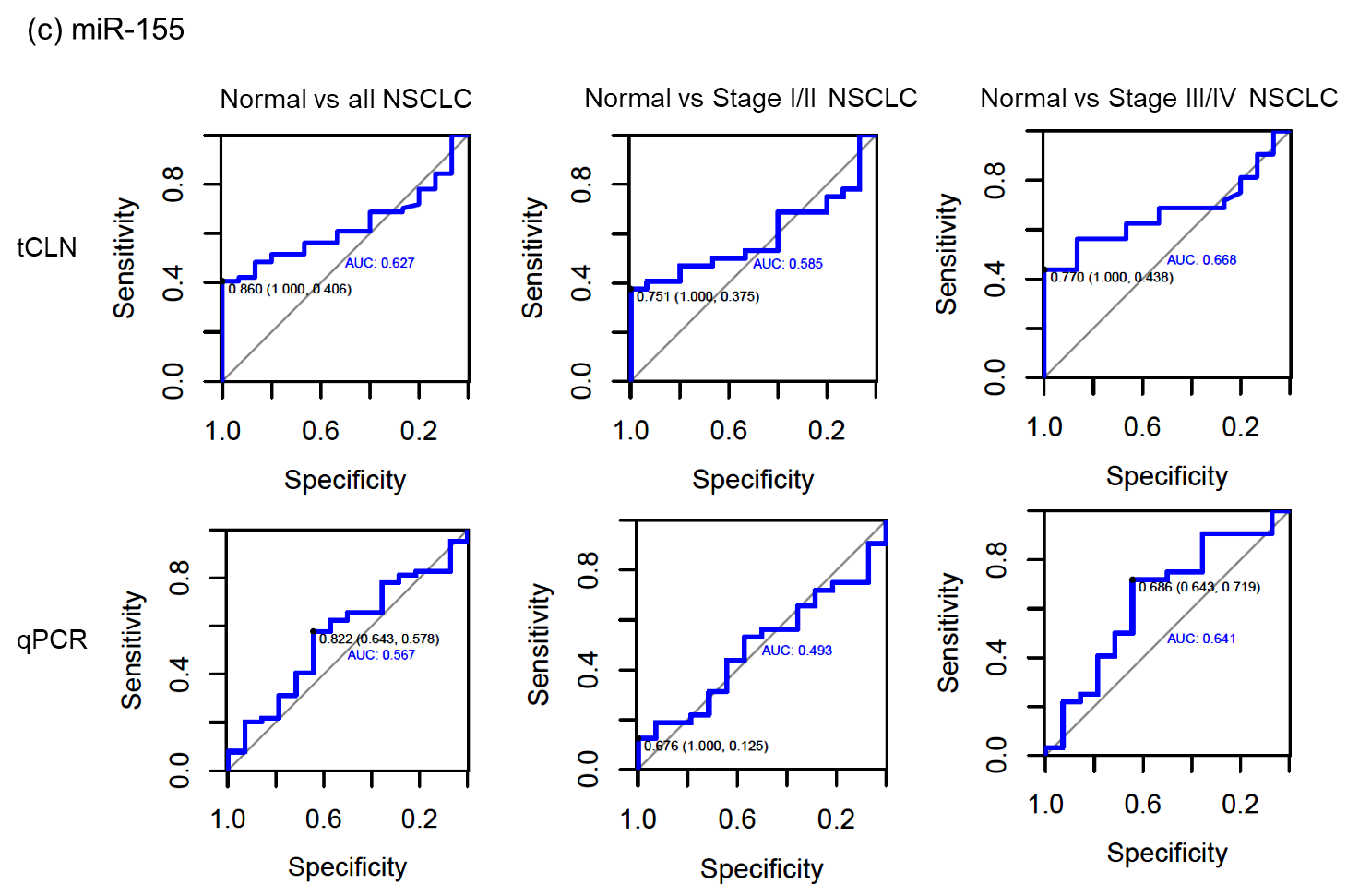
**

**
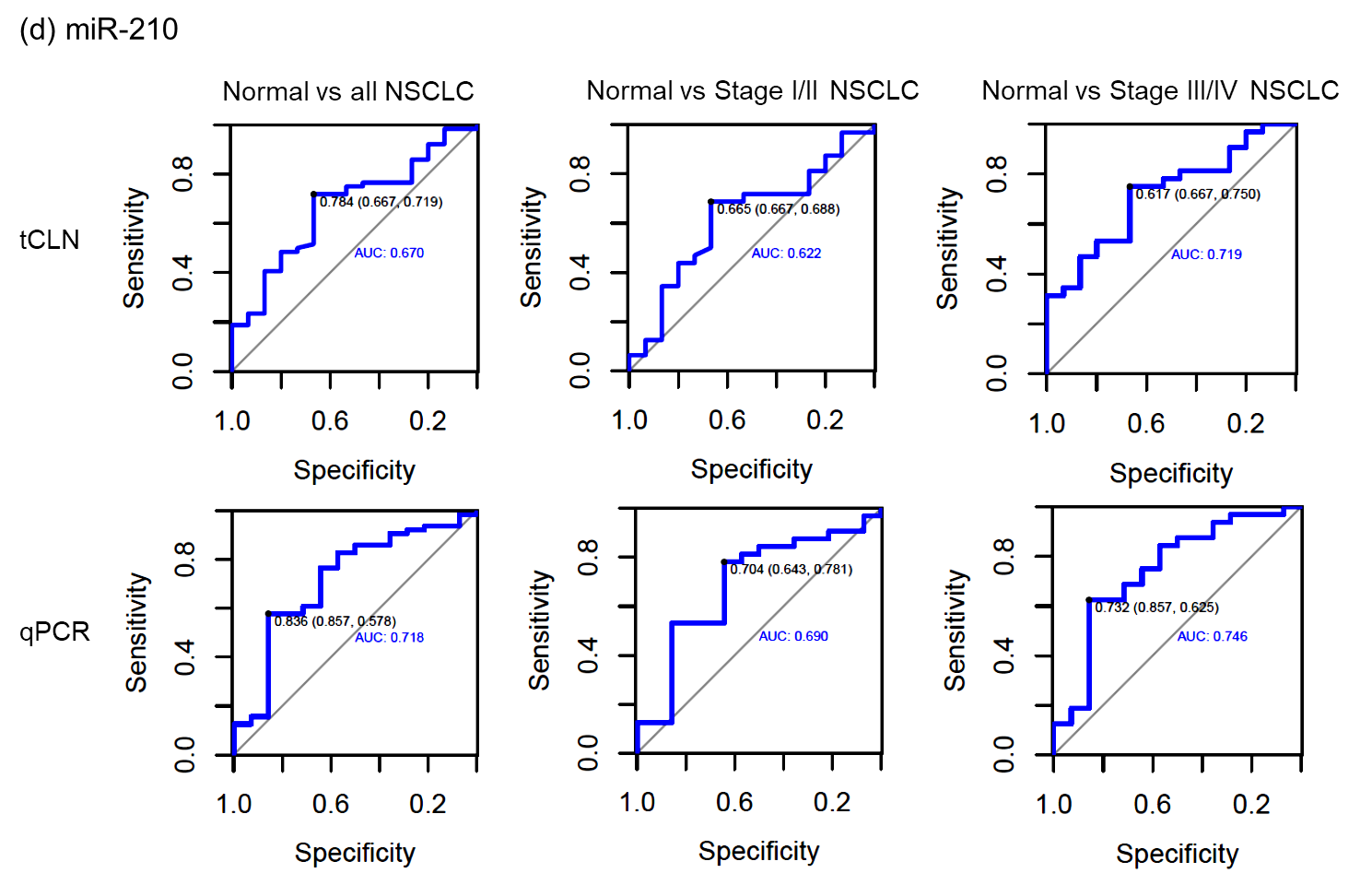
**

**
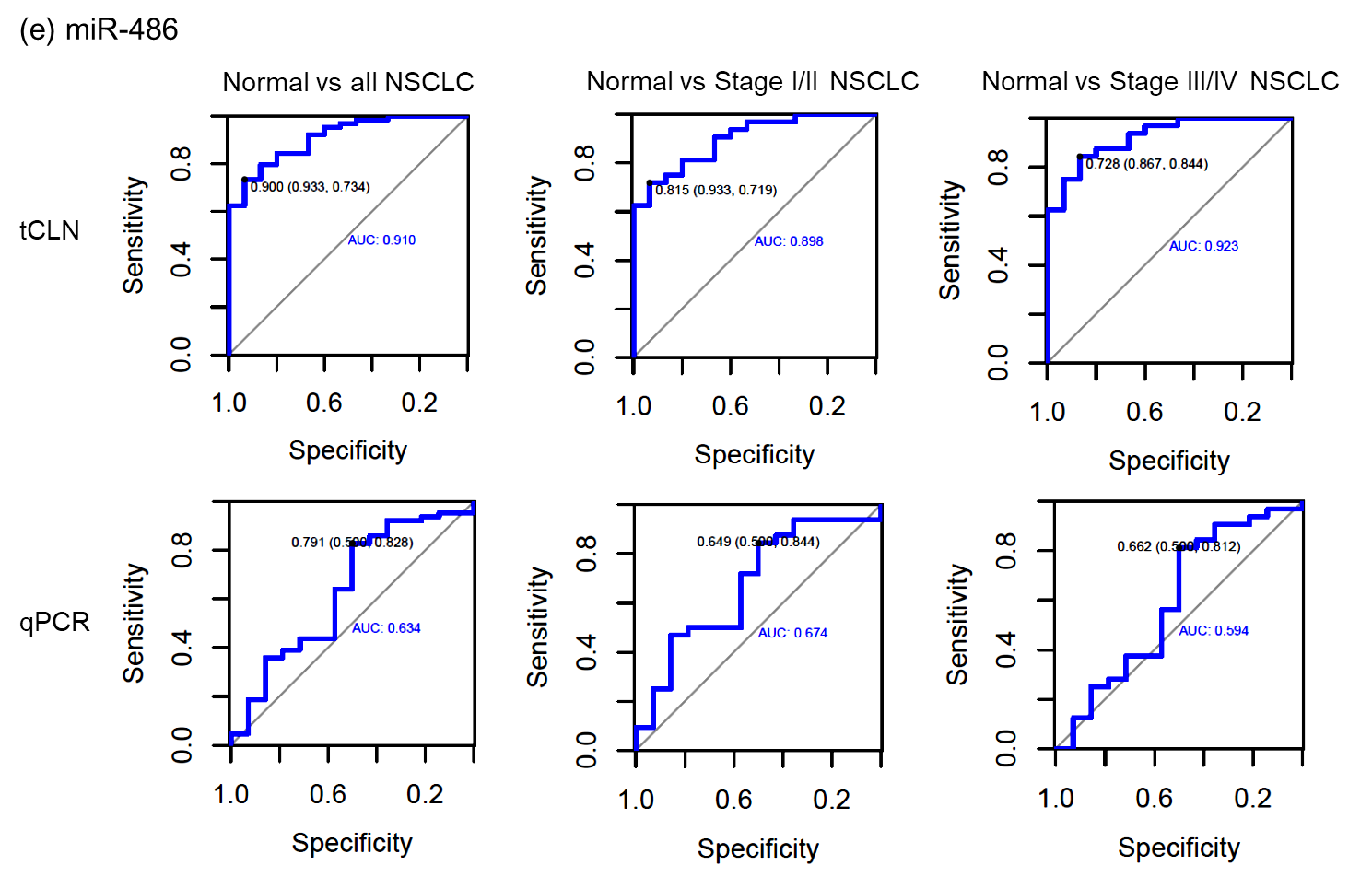
**
